# 

**Published:** 1920-06-12

**Authors:** 


### Forthcoming Events.

PADDINGTON GREEN CHILDREN'S HOSPITAL.—Thirty-seventh Annual Meeting, Thursday, JUNE 10, 5.30 P.M. Tea will be served at 4.30. F. J. Walker, Esq. (in the chair), supported by his Worship the Mayor of Paddington (Alderman H. G. Handover), Sir R. Melvill Beachcroft, Sir Henry P. Harris, K.B.E., D.L., M.P., Ald. W. G. Perring, M.P., Rev. Herbert Newell Bate, M.A., Rev. Dr. Haynes, Rev. W. G. Pennyman, and Rev. C. Knight.

OVERSEAS NURSING ASSOCIATION. — Annual Meeting, THURSDAY, JUNE 24, 3.30 P.M., at Norfolk House, St. James' Square, S.W., the Right Hon. Viscount Gladstone, G.C.B., in the chair. H.R.H. Princess Beatrice, Patroness of the Association, will honour the meeting by being present.

### Who Was Who.

A new reference book, "Who Was Who, 1897-1916," is about to be published by Messrs. A. and C. Black. This volume supplies biographical information of all dead celebrities whose names have appeared in "Who's Who" during the period mentioned. The price is to be one guinea net.

### Women's Holiday Fund.

THE object of this Society, which began its work in 1895, is to help the London working woman to have a week or a fortnight's holiday by the sea or in the country. Help is badly needed—for the lonely woman dependent on her own exertions for her livelihood, for the mother of a large family whose perplexities and cares increase as prices mount steadily upwards, and for the tired woman with her little baby, who is told by the doctor that all she needs is rest and change. The Committee informs us that some women are being sent away this year who have never had a holiday in their lives before—two of them are over sixty years old!—and they are now looking forward to it as a new and exciting experience. All applicants pay as much as they can towards their expenses, which are this year very heavy; but the Society has to meet about two-thirds of the total cost. If all holiday-makers will only allow their imagination full play, and will picture to themselves what it means not to have a holiday every year, or never to have one, they cannot help responding to the Committee's appeal, and will forthwith send a donation to the Secretary of the Women's Holiday Fund, 76 Denison House, 296 Vauxhall Bridge Road, S.W. 1.

### Medical Appointments.

Mr. T. Twistington Higgins, O.B.E., F.R.C.S., has been appointed surgeon (with charge of out-patients) at the Great Northern Central Hospital, Holloway.

The following appointments to the honorary medical and surgical staff of the Royal Victoria and West Hants Hospital, Bournemouth, have been made: Hon. assistant surgeon and surgeon in charge of the orthopædic department, Mr. Maitland B. Scott, O.B.E., F.R.C.S. (Ed.), L.R.C.P. (Lond.), M.R.C.S. (Eng.), of Shiplake, Bournemouth; hon. medical officer to the electro-therapeutical department, Miss Florence Stoney, O.B.E., M.D., B.S.

Only one application was received by the Breconshire County Council for the post of county medical officer and schools medical officer, vacant through the appointment of Dr. Colston Williams to a similar position under the Glamorgan County Council. The salary is £600 per annum, rising by yearly increments of £25 to £750, with travelling expenses. The solitary candidate, Mr. Herbert Davies, M.B., B.S. (Lond.), D.P.H., assistant schools medical officer under the Berkshire Education Committee, who is a native of Breconshire, appeared before the county authority on Friday and received the appointment.

### Passing Events and Topics.

### Proposed Hospital at Cairo.

A COMMITTEE has been appointed by the High Commissioner of Egypt to inquire into the proposal to erect a new British hospital in that city. An endowment of £E60,000, or £E4,000 annually, will be required.

### New Ward at Pontypool Hospital.

MR. JOHN PATON, J.P., of Wann Wern, Pontypool, recently opened a new ward, containing eight beds, in the Capel Wing of the Pontypool and District Hospital. In 1904 the number of patients in the hospital was 148, which had increased to 714 last year. Recent gifts to this hospital include a subscription of £620—their portion of the "Byng Millions"—from the Abersychan district ex-Service men, to perpetuate the memory of local men who fell in the war.

### A Successful Flag Day.

A RECENT Flag Day held by the League of the Roses (of which Miss M. F. Roby is chairman) in aid of the Great Northern Central Hospital realised £326 18s. 5d.

### The Theatrical Garden Party.

ORGANISED by Mr. Anslow Austin, 3 Middle Temple Lane, E.C. 4, in aid of the Actors' Orphanage, the party is to be held at the Royal Hospital Gardens, Chelsea, on June 22. Tickets are already obtainable from the Organising Secretary and at all the West End Box Offices and Agencies.

### Gifts and Bequests.

THE Great Northern Central Hospital has received a present of cut flowers from the King and Queen, and the Queen has sent a gift of wild flowers to Queen Charlotte's Hospital.

MR. A. E. HEAD, of Bristol, has bequeathed £500 to the Seamen's Hospital Society and £200 each to the Reigate and Redhill Cottage Hospital and the Dorset County Hospital.

SMITH'S KENSINGTON CHARITY have given £500 to the Royal Dental Hospital and £100 to the Finchley branch of the National Hospital for the Paralysed and Epileptic.

THE Trustees of the Zunz Bequest have granted £400 to the Chelsea Hospital for Women and £250 to the National Hospital for the Paralysed and Epileptic.

MRS. L. S. DIGBY, of North Runcton, Norfolk, left £1,100 for charitable and religious purposes.

MR. C. H. EIGER, of the Stock Exchange, left £1,000 to the London Hospital and £250 to the Jews' Hospital for Incurables at South Tottenham.

THE residue of the estate of Mr. F. J. Warner, of Brighton, valued at about £20,000, is to be divided between the London Hospital and Middlesex Hospital.

THE WEST LONDON HOSPITAL has received an award of £400 from the Trustees of the Zunz Bequest, and a donation of £500 from Smith's (Kensington Estate) Charity.

THE late Mr. J. C. Harvey has bequeathed £500 to the Royal Victoria and West Hants Hospital, Bournemouth.

MR. F. W. R. SKEY, of Maida Vale, has left £2,000 each to the Prince of Wales's Fund and St. Bartholomew's Hospital, and £1,000 to the Essex County Hospital. The residue of the estate is left to St. Dunstan's.

HULL ROYAL INFIRMARY has been left £3,000 by Mr. George Martinson, a solicitor, of Hull.

### Honour for Hospital Pharmacist.

MR. F. ALMOND HOCKING, B.Sc., chief pharmacist of the London Hospital, has been appointed an examiner by the Pharmaceutical Society of Great Britain for the statutory qualifying examination of pharmacist, subject to the approval of the Privy Council. It is noteworthy that the services of several prominent hospital pharmacists are used for this purpose, among whom may be mentioned Mr. Jennings (St. Thomas's), Mr. Hampshire (University), Mr. Peck (Great Ormond Street), Mr. Taylor (Bristol Royal), and Mr. Marsden (Liverpool Royal).

### British Pharmaceutical Conference.

### PROGRAMME AT LIVERPOOL.

THE British Pharmaceutical Conference will hold its annual meeting at Liverpool on July 19 and following days, and an attractive programme is promised by the local committee.

Papers are to be contributed on pharmaceutical matters, and a series of visits to places of note in Liverpool and neighbourhood has been arranged, with a full day's motor trip to North Wales.

The social side of the conference has been curtailed during the war, and at this year's gathering it is hoped to revive it.

Many hospital pharmacists are active workers on the Conference Committee. Mr. C. H. Hampshire, B.Sc., chief pharmacist at University College Hospital, is one of the joint secretaries with Mr. R. R. Bennett, who was formerly at "University," and now has an important post with a wholesale firm of druggists.

